# Potential gains in reproductive-aged life expectancy if maternal mortality were eradicated from the Kintampo districts of Central Ghana

**DOI:** 10.1186/s12884-019-2515-0

**Published:** 2019-10-23

**Authors:** Sulemana Watara Abubakari, Ayaga Agula Bawah, Ernest Obed Nettey, Edward Anane Apraku, Charles Zandoh, Seeba Amenga-Etego, Kwaku Poku Asante, Seth Owusu-Agyei, Delali Margaret Badasu

**Affiliations:** 1 Kintampo Health Research Centre, Ghana Health Service, P. O. Box 200, Kintampo, Ghana; 2 Regional, Institute for Population Studies, University of Ghana, P. O. Box LG 96, Legon, Ghana; 3 Institute of Health Research, University of Health & Allied sciences, Ho, Volta Region Ghana

**Keywords:** Maternal mortality, Causes of death, Reproductive-aged life expectancy, Women of reproductive age

## Abstract

**Background:**

Almost 99% of pregnancy or childbirth-related complications globally is estimated to occur in developing regions. Yet, little is known about the demographic impact of maternal causes of death (COD) in low-and middle-income countries. Assuming that critical interventions were implemented such that maternal mortality is eradicated as a major cause of death, how would it translate to improved longevity for reproductive-aged women in the Kintampo districts of Ghana?

**Methods:**

The study used longitudinal health and demographic surveillance data from the Kintampo districts to assess the effect of hypothetically eradicating maternal COD on reproductive-aged life expectancy by applying multiple decrement and associated single decrement life table techniques.

**Results:**

According to the results, on the average, women would have lived an additional 4.4 years in their reproductive age if maternal mortality were eradicated as a cause of death, rising from an average of 28.7 years lived during the 2005-2014 period to 33.1 years assuming that maternal mortality was eradicated. The age patterns of maternal-related mortality and all-cause mortality depict that the maternal-related mortality is different from the all-cause mortality for women of reproductive age.

**Conclusion:**

This observation suggests that other COD are competing with maternal mortality among the WRA in the study area and during the study period.

## Background

Women in every country are reported to have a longer life expectancy at birth than men [1]. However, there is little understanding of how maternal mortality influences improvement in women’s life expectancy. Understanding the effects of maternal mortality on life expectancy of women is particularly important in low-and middle-income countries (LMICs) where 99% of maternal deaths occur [1]. Despite the attention that maternal mortality has received over the past three decades, researchers have paid little attention to the measurement of the demographic impact of maternal mortality in LMICs, especially, in sub-Saharan Africa (SSA) where the burden of maternal mortality is highest.

The majority of the countries in SSA region have maternal mortality ratios (MMR) of over 300 maternal deaths per 100,000 live-births [2, 3]. Maternal mortality in excess of 300 maternal deaths per 100,00 live births is classified as high [3]. SSA, compared to other developing regions of the world, has a very high MMR [4, 5]**.** It has a point-estimate of 546 compared to 187 in Oceania, 176 in Southern Asia and 110 in South-eastern Asia. Moreover, the lifetime risk of maternal mortality is estimated at 1 in 36 in SSA compared to 1 in 2300 in Eastern Asia and 1 in 1100 in Caucasus and Central Asia. In the high-income settings, the lifetime risk is estimated at 1 in 4900 which contrasts sharply with the risk in SSA. Among the 20 countries with the highest maternal mortality ratios, only Afghanistan is not in SSA region [4]. Using the 1990 to 2013 global burden of disease (GBD) data, there would be 184,100 maternal deaths globally in 2030. SSA is projected to account for 38 out of 53 countries that are estimated to have MMR of more than 100 maternal deaths per 100,000 live-births which fall short of the less than 70 maternal deaths per 100,000 live births SDG 3.1 target [5].

Although efforts intensified to reduce maternal mortality since the launch of the “Safe Motherhood” initiative in the late 1980s, progress has been rather slow, the result being continuous and persistent high levels of mortality during pregnancy or childbirth by women. Questions as to how many woman-lives could be saved during pregnancy or at childbirth remain, particularly in SSA. This study is intended to bridge the gap in the literature by assessing the total number of female person-years that might be saved during the reproductive period if maternal mortality were eradicated from the population of women of reproductive age (WRA).

A longitudinal health and demographic surveillance data from the Kintampo Health Research Centre (KHRC) of Ghana were used. To answer the question “how many woman-lives could be saved if deaths due to pregnancy-related or childbirth were averted, we used multiple decrement and associated single decrement life table techniques [6, 7] to estimate woman-life years saved. We conducted the analysis on the assumption that the mortality conditions for the period 2005-2014 for WRA remained unchanged.

## Methods

### Data source

Data for this study came from the Kintampo Health and Demographic Surveillance System (KHDSS) of KHRC. KHDSS covers the Kintampo districts of the Brong Ahafo Region of Ghana [8–10]. One of the key characteristics of the KHDSS is its longitudinal approach to the measurement of demographic and health variables. Repeated visits by fieldworkers at regular intervals to all residential units in the KHDSS area allow them to collect a prescribed set of attribute data on registered subjects, who are consistently and uniquely identified. All deaths that occur in the study area are captured using death registration forms and information subsequently entered into a death registration database which forms part of the linked database of the KHDSS.

To ascertain the cause of death, a validated verbal autopsy (VA) instrument was used to elicit information on circumstances and conditions that prevailed as observed by the primary caregiver of the deceased, from when the individual fell sick until her death. Verbal autopsy involves interviewing relatives or caregivers who were closely associated with the deceased during the period leading to her death [7, 11]. Two physicians independently reviewed the VA questionnaire and independently assign a probable cause of death, using the 10th revision of the International Statistical Classification of Diseases and Related Health Problems (ICD-10). If there is agreement between the two independently assigned cause, then it is designated as the cause of death. However, if there is no agreement between the two physicians, a third physician is asked to examine the case and if there is no agreement, then the cause of death for that case is considered unknown or indeterminate [9, 12, 13].

### Data management

Prior to data processing, data verification and consistency checks were conducted to ensure completeness and consistency. In addition, basic demographic checks such as age-specific mortality rates were conducted to ensure that the mortality profile exhibited by the data is consistent with what is expected of a developing country like Ghana. Data processing was carried out using Microsoft Visual Foxpro (version 9.0). All VA questionnaires were double-entered and verified with an automated range and consistency checks.

### Estimation procedure

This study defines maternal deaths to include only direct or obstetric COD as the death of a woman while pregnant or within 42 days after the termination of the pregnancy, irrespective of the duration and site of the pregnancy. Reproductive-aged life expectancy (RALE) which is estimated in this study refers to the average number of years to be lived by a woman during her reproductive age period (15-49 years), is calculated similar to that of overall life expectancy but limited to the ages from 15 to 49 years, using age 15 as the radix of the life table in this case. RALE is suitable for this study because maternal mortality is conditioned on survival to age 15 and takes into account all causes of mortality [14, 15].

The effect of maternal COD on overall mortality was evaluated by estimating the number of person-years that could be saved assuming maternal COD were eradicated as a major cause of death among females in the Kintampo districts. The impact of maternal COD is estimated using multiple-decrement and associated single-decrement life-table techniques [6, 7, 12]. These methods estimate the net effect of competing risks from different causes. To undertake this analysis, we isolated all deaths due to maternal causes (pregnancy or childbirth related deaths) and lumped all other causes other than the identified maternal deaths into an alternate category. This is important because of the competing risks analysis (maternal versus all other causes). Competing risks analysis operates under the assumption that different causes operate independently of each other to affect mortality. An algebraic explication of the estimation procedure as outlined by Bawah and Binka (2007) is shown in the Appendix.

### Data limitations

The influence of mortality from maternal COD may not have been estimated accurately since not all deaths recorded by the KHDSS had successful verbal autopsy interviews. However, this is expected to be self-selected and, therefore, should not have major effects on this study. In addition, a proportion of the cases with successful interviews were coded as “cause of death not determined”. This is also expected to be random. Furthermore, there are possibilities of wrongly assigning of COD but again, this is not expected to significantly affect the results since they are very likely to be self-selected. Moreover, child deaths are more likely to be missed than adults. Babies who die a few days or weeks after delivery are usually not reported by some family members who do not count them as human beings. Individuals considered for this study were all adults who were less likely to have been missed under such circumstances.

## Results

Table 1 presents a general and multiple-decrement life table for WRA in the Kintampo districts from 2005 to 2014. We restricted the life table to WRA because of our interest in computing reproductive life years saved. For the period 2005-2014, a total of 1259 deaths occurred among women in their reproductive ages (15-49 years), of which 74 women were estimated to have died from maternal-related causes as defined earlier. By age group, 116 women died in 15-19 age group, 163 in the 20-24 age group, 213 in the 25-29 age group, and so on from all-cause mortality. The distribution of the 74 maternal mortality related deaths by age group within the same age groups were 11 within the age-group 15-19, 14 in the 20-24 age group, 25 in the 25-29 age group, 11 in the 30-34 age group, 7 in the 35-39 age group, 4 in the 40-44, and 2 in the 45-49 age group. Constructing a single decrement life table for all deaths irrespective of cause results in a life expectancy of 28.7 for women within the reproductive ages (ages 15-49 years).

**Table 1:** A general and multiple-decrement life table for WRA, KHDSS (2005 to 2014)

Age x	PYO	D^All^	D^MM^	_n_a_x_	_n_m_x_	_n_q_x_	_n_p_x_	l_x_	_n_d_x_	_n_L_x_	T_x_	e_x_	_n_q_x_^MM^	_n_d_x_^MM^	l_x_^MM^	_n_m_x_^MM^
15-19	70,605	116	11	2.478	0.0016	0.0082	0.9918	100,000	818	497,937	2,865,517	28.7	0.0008	78	703	0.0002
20-24	60,178	163	14	2.711	0.0027	0.0135	0.9865	99,182	1335	492,853	2,367,580	23.9	0.0012	115	626	0.0002
25-29	52,484	213	25	2.605	0.0041	0.0201	0.9799	97,847	1966	484,525	1,874,727	19.2	0.0024	231	511	0.0005
30-34	46,333	222	11	2.565	0.0048	0.0237	0.9763	95,881	2271	473,874	1,390,202	14.5	0.0012	113	280	0.0002
35-39	39,707	227	7	2.534	0.0057	0.0282	0.9718	93,610	2639	461,544	916,327	9.8	0.0009	81	168	0.0002
40-44	33,456	178	4	2.470	0.0053	0.0262	0.9738	90,971	2388	448,816	454,784	5.0	0.0006	54	86	0.0001
45-49	26,742	140	2	2.606	0.0052	0.0259	0.9741	88,584	2290	5968	5968	0.1	0.0004	33	33	0.0001
Total	329,505	1259	74	–	–	–	–	–	–	–	–	–	–	703	–	–

Data source: Kintampo HDSS (2005-2014)

Notes:

PYO = Person years of observation

MM = Maternal causes of death

_n_a_x_ = Average number of person-years lived in the interval by those who have died in the interval

_n_m_x_ = Mortality rate for people in age group x to x + n

_n_q_x_ = Probability of dying between ages x and x + n

_n_p_x_ = Probability of surviving between ages x and x + n

l_x_ = Number surviving at each age

_n_d_x_ = Number of deaths between ages x and x + n

_n_L_x_ = Person-years lived between ages x and x + n

T_x_ = Person-years lived beyond age x

e_x_ = Life expectancy at age x

To estimate the impact of maternal mortality on overall mortality for women in their reproductive ages, we isolate the 74 deaths due to maternal mortality and estimated a multiple decrement life table to enable us answer the question ‘*how many women who reached the reproductive age would eventually die from maternal mortality related causes?’* So, assuming a hypothetical situation where 100,000 women survived from birth to age 15 and are subjected to the age-specific mortality conditions of the period, about 703 of them would eventually die from maternal mortality by the time they attain age 49 (see third to the last column of Table 1). In Table 2, we estimate the gains in RALE assuming maternal mortality was eradicated. To do this, we used cause-deleted life table analysis (Table 2). The resultant RALE is estimated at 33.1, suggesting a gain of 4.4 years, from a reproductive age life expectancy of 28.7 to 33.1 years, during the period.

**Table 2:** Associated single-decrement life table for COD other than maternal causes for KHDSS from 2005 to 2014

Age x	l_x_	_n_p_x_	R^-MM^	P^-MM^	l_x_^-MM^	_n_q_x_^-MM^	_n_d_x_^-MM^	_n_q_x_/ _n_q_x_^-MM^	_n_a_x_^-MM^	_n_m_x_^-MM^	_n_L_x_^-MM^	T_x_^-MM^	e_x_^-MM^
15-19	100,000	0.9918	0.9052	0.9926	100,000	0.0074	741	1.1043	2.4969	0.0015	498,146	3,306,877	33.1
20-24	99,182	0.9865	0.9141	0.9877	99,259	0.0123	1222	1.0933	2.6705	0.0025	493,450	2,808,731	28.3
25-29	97,847	0.9799	0.8826	0.9822	98,037	0.0178	1741	1.1316	2.6133	0.0036	486,031	2,315,282	23.6
30-34	95,881	0.9763	0.9505	0.9775	96,296	0.0225	2169	1.0515	2.5799	0.0046	476,233	1,829,251	19.0
35-39	93,610	0.9718	0.9692	0.9727	94,128	0.0273	2573	1.0314	2.5147	0.0055	464,244	1,353,018	14.4
40-44	90,971	0.9738	0.9775	0.9743	91,555	0.0257	2350	1.0227	2.4892	0.0052	451,875	888,774	9.7
45-49	88,584	0.9741	0.9857	0.9745	89,205	0.0255	2274	1.0143	0.9857	0.0052	436,899	436,899	4.9

Data source: Kintampo HDSS (2005-2014)

Note:

MM = Maternal causes of death

R^−MM^ = the proportion of deaths due to all causes other than maternal causes

_n_a_x_ = Average number of person-years lived in the interval by those who have died in the interval

_n_m_x_ = Mortality rate for people in age group x to x + n

_n_q_x_ = Probability of dying between ages x and x + n

_n_p_x_ = Probability of surviving between ages x and x + n

l_x_ = Number surviving at each age

_n_d_x_ = Number of deaths between ages x and x + n

_n_L_x_ = Person-years lived between ages x and x + n

T_x_ = Person-years lived beyond age x

e_x_ = Life expectancy at age x

**Fig. 1:**
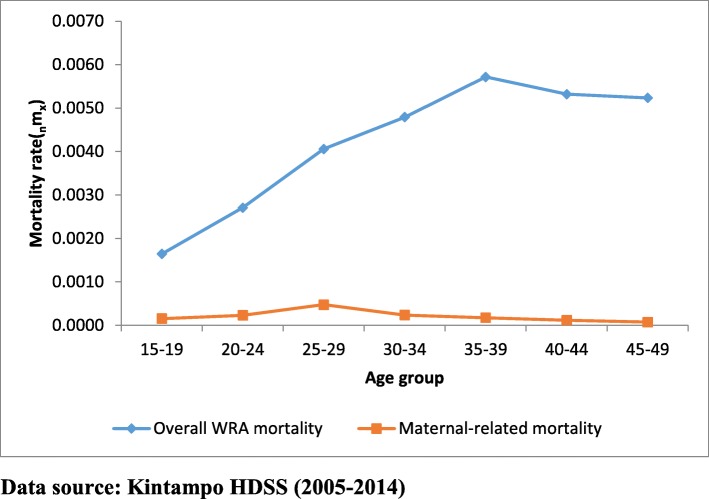
Age pattern of maternal causes for overall mortality and maternal causes-specific mortality

Figure 1 shows the age pattern of COD for overall mortality and maternal-related mortality for WRA. The age pattern of maternal-related mortality is not similar to that of all-cause mortality for WRA. The maternal-related mortality peaked at 25-29 years and declined steadily after that while the overall mortality for WRA peaked at 35-39 years and began a downward trend after that but remained stable from age 40 to 49. This suggests that causes other than maternal-related death are the major COD among the WRA in the study area and during the study period.

**Fig. 2:**
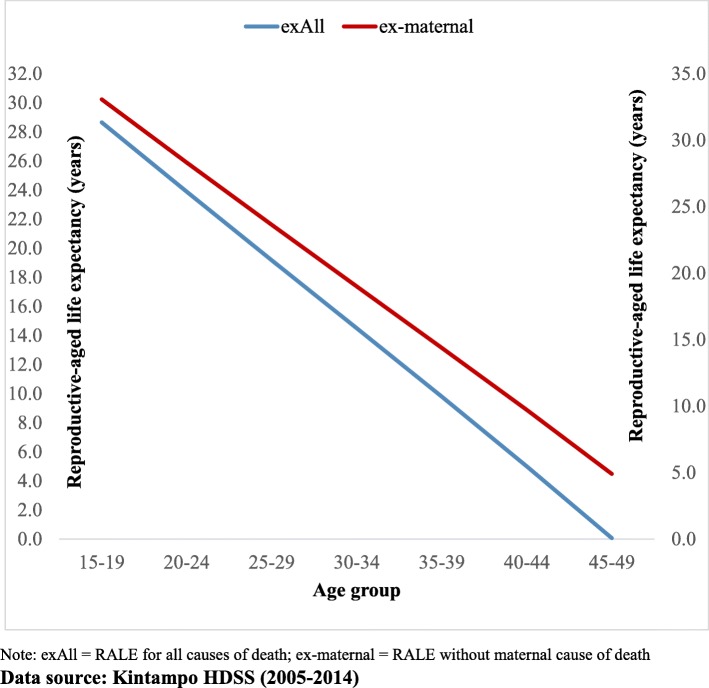
Comparison of estimated life expectancy for WRA with all-cause mortality and without maternal COD, KHDSS (2005-2014)

Figure 2 shows the RALE at every age when all COD among WRA are put together and the corresponding RALE if maternal mortality were removed. At every age, RALE increased if maternal mortality were averted. The rise in life expectancy is relatively more evident at the older ages of life, when the force of mortality is higher than at other ages.

## Discussions

This paper sought to estimate potential improvement in RALE if maternal mortality had been eradicated or reduced to insignificant limits such that it is no longer of public health concern in the study area. The results show that the average number of years lived between ages 15 and 49 increased from 28.7 to 33.1 years in the absence of maternal mortality, a probable gain of about 4.4 years. This provides an average annual gain in years of about 0.4 per year over the ten-year study period (2005-2014).

RALE gains of 0.4 years per annum observed in the present study is quite consistent with findings from both the Global North and Global South. For instance, Canudas-Romo et al. (2014) reported an annual gain of 0.5 years due to elimination of maternal mortality in high-income settings during the first half of the twentieth century. Similarly, they reported an annual gain of 0.6 years, assuming maternal mortality was eliminated in low and middle-income countries between 2000 to 2011 [12]. Evidence of consistent reductions in maternal mortality makes the finding in the present study quite plausible [13, 14].

It is documented that maternal mortality declined by 1.5% per year between 1990 and 2015 globally [3]. The decline was largely as a result of global efforts in the past three decades [15]. Improvement in maternal mortality accelerated after the introduction of MDG 5A in 2000 which aimed to reduce the global MMR by at least 75% by 2015 [13, 14]. It is observed from the present study that causes other than maternal-related mortality are the major COD among the WRA in the study area and during the study period. This observation is probably due to the current evidence of reduction in maternal mortality as a competing cause of death.

Since the additional survival time of 0.4 years takes place during the most productive ages of women, their contributions to the socio-economic development of families, labour force, communities, districts and the country as a whole would be invaluable if maternal mortality were eradicated. Households that experience a maternal death may suffer reduced economic output due to the loss of mothers and wives in the household. The loss of a mother in the household may result in changes in household duties and management that affect children’s schooling, nutrition and supervision [12] and overall care.

It is important to note that RALE would increase at every age if maternal mortality were eradicated from this population. The results obtained from the study underscore the need for policy and intervention to focus on all WRA and most especially on those who are older since averting maternal deaths disproportionately lead to a greater rise in RALE among the older WRA who experience a higher force of mortality.

Maternal mortality is relatively a rare event compared to the other COD among WRA and yet influences the extent of the gain in RALE. It is reported that in developed countries, maternal mortality is underestimated by about a third. The inaccuracies are much higher in LMICs [12, 16]. However, wider usage of VA for mundane national health information systems could potentially enhance the availability of essential and reliable data on COD for disease control programmes.

## Conclusion

RALE would have improved among women in the Kintampo districts if maternal mortality was eradicated from this population, given the age-specific mortality rates for the period 2005-2014. The number of years gained would have been quite considerable, an average of about 0.4 years per year given that the average number of years lived from age 15 to 49 would have increased from 28.7 to 33.1 years in the absence of maternal mortality.

The increase in RALE suggested by the results may be achieved eventually if efforts aimed at reducing maternal mortality are enhanced. There is also the need to investigate other COD that might be competing with maternal death as far as the population of WRA in the study area is concerned since the evidence from this analysis points to competition from other COD.

## Data Availability

The datasets used and/or analysed during the current study are available from the corresponding authour on reasonable request.

## Appendix

### Estimation procedure for multiple decrement and associated single decrement life tables

In the analysis of causes of death, the force of the mortality function from different causes is additive because disentangling precisely the effects of other causes of death is difficult, especially in settings where precise measurement is not possible. Thus, the sum of the different causes is equal to all causes combined as represented in Eq. (1) thus:

1 $$ \mu (x)=\sum \limits_{i=1}^I{\mu}_i(x), $$

where μ(*x*) is the force of mortality from all causes combined and parameters μ_*i*_(*x*) refer to the death rate for the *i*th cause of death. This implies that the rates of decrements are also additive:

2 $$ {{}_nm}_x=\sum \limits_{i=1}^i{{}_nm}_{xi}, $$

where _*n*_*m*_*x*_ is the rate of decrement from all causes and _*n*_*m*_*xi*_ in this case is the rate of decrement from maternal causes of death.

Considering the basic relationship between mortality rates(_*n*_*m*_*x*_) and the probability of dying (_*n*_*q*_*x*_) as shown in the conventional life table, the transformation of the rates to probabilities of dying is shown in the following equation as:

3 $$ {{}_nq}_x=\frac{n_n{m}_x}{1+\left(n-{{}_na}_x\right){{}_nm}_x}, $$

where _*n*_*a*_*x*_ is defined as the average number of person-years lived in the interval *x* to *x + n* by those who died in the interval. This relationship extends to multiple-decrement processes as follows:

4 $$ {{}_nq}_{xi}=\frac{n_nm{}_{xi}}{1+\left(n-{{}_na}_x\right)\left({{}_nm}_{xi}+{{}_nm}_{x,-1}\right)}, $$

where _*n*_*m*_*xi*_ and _*n*_*m*_*x,–i*_ represent decrement rates from maternal and all other causes other than maternal combined, respectively. Data concerning the causes of death by age and the corresponding number of person-years by the same sub-categories define the probabilities of dying at each age (_*n*_*q*_*x*_), by cause of death. However, obtaining the _*n*_*a*_*x*_ values is often difficult. Therefore, different techniques are employed to estimate the _*n*_*a*_*x*_ values. First, it is assumed that those who died in the interval on average lived halfway through the interval. Based on this assumption, an initial value of 2.5 is adopted for all age groups with an interval of 5 years. For the younger than 1-year and 1–4-year age groups, a procedure suggested by Coale and Demeny is adopted (Bawah and Binka, 2007).

Using the _*n*_*a*_*x*_ values of 2.5 in the _*n*_*m*_*x*_→ _*n*_*q*_*x*_ conversion formula, _*n*_*q*_*x*_ values are estimated first and the values are used to obtain *ndx* (the number of deaths between age *x* and *x + n*) in a life table. These _*n*_*d*_*x*_ estimates are plugged into the iteration formula below to obtain new sets of _*n*_*a*_*x*_ values. These values are subsequently re-introduced into the _*n*_*m*_*x*_→_*n*_*q*_*x*_ conversion formula to re-estimate new _*n*_*d*_*x*_ values, which are re-introduced in the iteration formula to obtain a new set of _*n*_*a*_*x*_ values. This process is repeated until stable estimates of _*n*_*a*_*x*_ are achieved (Bawah and Binka, 2007). The iteration equation used is specified as follows:

5 $$ {{}_na}_x=\frac{-\frac{n}{24}{{}_nd}_{x-n}+\frac{n}{2}{{}_nd}_x+\frac{n}{24}{{}_nd}_{x+n}}{{{}_nd}_x}. $$

The stable _*n*_*a*_*x*_ values then are used to generate a life table for females in the Kintampo HDSS area through the basic _*n*_*m*_*x*_ → _*n*_*q*_*x*_ conversion formula. With the overall life table generated, the probability of dying from maternal causes of death (_*n*_*q*_*xi*_) is estimated, by applying the proportion of deaths that are due to maternal causes of death to the overall probabilities of dying for each age, _*n*_*q*_*x*_, as follows:

6 $$ {{}_nq}_{xi}={{}_nq}_x\cdot \frac{{{}_nD}_{xi}}{{{}_nD}_x}, $$

where _*n*_*q*_*xi*_ and _*n*_*D*_*xi*_ represent the probability of dying from maternal causes of death and the observed number of deaths from maternal causes of death, respectively. The above relationship assumes that the observed death rates for maternal causes of death (_*n*_*M*_*xi*_) are equal to the life-table death rates for maternal causes of death (_*n*_*m*_*xi*_), that is, _*n*_*M*_*xi*_ *=*_*n*_*m*_*xi*_.

Estimating the contribution of mortality from maternal causes of death to overall mortality also allow to estimate the effect of eliminating maternal causes of death through “cause-deleted” lifetable analysis (Bawah and Binka, 2007). If maternal-related mortality were eliminated as a cause of death, survival at age interval *x* to *x* + *n*, will be represented as:

7 $$ {}_nP{}_{x,-i}={{}_np}_x\left(\frac{{{}_nD}_{xi}}{{{}_nD}_x}\right). $$

The approach described above assumes that the force of mortality function from each cause is proportional to all causes combined in the interval *x* to *x + n* and constant throughout the interval (Arriaga, 1984). The _*n*_*a*_*x*_ values for the associated single decrement life table were obtained using the following formula for all age groups except the first two and the last:

8 $$ {{}_na}_{x,-1}=n+{R}^i\frac{{{}_nq}_x}{{{}_nq}_{x,-1}}\left({{}_na}_x-n\right), $$

where _*n*_*a*_*x*_*,*_*−i*_ refers to the average number of person-years lived by those dying in the interval from all causes other than maternal-related death, and *Ri* represents the proportion of deaths due to maternal-related mortality. For the other age groups, the iteration procedure used for estimating the _*n*_*a*_*x*_ values in the parent life table is used.

## Abbreviations

COD: Causes of Death
GBD: Global Burden of Disease
ICD-10: the 10th revision of the International Statistical Classification of Diseases and Related Health Problems
IEC: Institutional Ethics Committee
KHDSS: Kintampo Health and Demographic Surveillance System
KHRC: Kintampo Health Research Centre
LMICs: Low- and Middle-Income Countries
MDG: Millennium Development Goal
MMR: Maternal Mortality Ratio
PCVA: Physician Coding Verbal Autopsy
RALE: Reproductive-aged Life Expectancy
SDGs: Sustainable Development Goals
SSA: Sub-Saharan Africa
UNFPA: United Nations Population Fund
UNICEF: United Nations Children’s Emergency Fund
VA: Verbal Autopsy
WHO: World Health Organization
WRA: Women of Reproductive Age
